# Effects of *Litsea cubeba* (Lour.) Persoon Essential Oil Aromatherapy on Mood States and Salivary Cortisol Levels in Healthy Volunteers

**DOI:** 10.1155/2020/4389239

**Published:** 2020-07-26

**Authors:** Chaiyavat Chaiyasut, Bhagavathi Sundaram Sivamaruthi, Jakkrit Wongwan, Kanyarat Thiwan, Wandee Rungseevijitprapa, Areewan Klunklin, Wipada Kunaviktikul

**Affiliations:** ^1^Innovation Center for Holistic Health, Nutraceuticals and Cosmeceuticals, Faculty of Pharmacy, Chiang Mai University, Chiang Mai 50200, Thailand; ^2^Department of Pharmaceutical Chemistry and Technology, Faculty of Pharmaceutical Sciences, Ubon Ratchathani University, Ubon Ratchathani 34190, Thailand; ^3^Faculty of Nursing, Chiang Mai University, Chiang Mai 50200, Thailand

## Abstract

Aromatherapy is one of the complementary therapies to improve health. The aromatic essential oils have been used in the treatment procedure through inhalation of essential oil vapor, massage, and herbal bathing. *Litsea* species are generally used in traditional medicine, and *Litsea cubeba* (Lour.) Persoon is a potent fumigant plant, used in cosmetics and foods as essence. The chemical composition of the essential oil of different parts of *L. cubeba* has been found to be varied. *L. cubeba* essential oil (LEO) is known for the treatment of cognition-associated discomforts. The current study assessed the impact of inhalation of LEO on mood states and salivary cortisol levels of healthy people. Fifteen healthy volunteers were involved in the study. The Profile of Mood States (POMS) Questionnaire and ELISA methods were employed to determine the mood states and salivary cortisol level, respectively. (-)-*β*-pinene, *β*-citral, *cis*- and *trans*-citral, citronellal, limonene, linalool, and 6-methyl-5-hepten-2-one were detected in LEO by GC-MS analysis. The heart rate and blood pressure were not affected significantly during LEO exposure. The inhalation of LEO significantly improved the total mood disturbance and reduced the confusion among the healthy human subjects. LEO inhalation reduced the salivary cortisol level at a notable level. The results of the current study warrant further studies on the beneficial effect of LEO aromatherapy in healthy and diseased subjects to uncover the therapeutic nature of the *L. cubeba* plant.

## 1. Introduction

Aromatherapy is one of the complementary therapies to improve health. The aromatic essential oils have been used in the treatment procedure through inhalation of essential oil vapor, massage, and herbal bathing. Aromatherapy helps to improve the quality of the lifespan of chronic patients. For instance, the lavender oil-based aromatic massage therapy and *Rosa damascene Mill* essential oil therapy improved the sleep quality of cardiac patients [1, 2].

*Litsea* is one of the diverse genera, which contains 200–400 species of the Lauraceae family distributed across the tropical and subtropical regions. *Litsea* species are commonly used in the traditional medicine of several nations for thousands of years [3]. *Litsea cubeba* (Lour.) Persoon is an aromatic plant widely scattered in Australia, America, Japan, China, and Southeastern Asia [4]. *L. cubeba* is a potent fumigant plant [5] and also used in cosmetics and foods as essence [6, 7]. The chemical composition of the essential oil of different parts of *L. cubeba* has been reported [7, 8]. It is proved that the chemical composition of the essential oil differed among the different parts of the plant and based on extraction methods. Citral B (neral), *β*-terpinene, and *β*-phellandrene are mainly present in the essential oil of *L. cubeba* extracted from roots and fruits, flowers, and leaves and stems, respectively [7]. *L. cubeba* have been reported for its antimicrobial [9, 10], anti-inflammatory [11], anticancer [12], antioxidative [13], and insecticidal activities [14].

Though *L. cubeba* essential oil (LEO) is known for the treatment of cognition-associated discomforts [15], the effect of LEO on the mood state and the impact of LEO on the salivary cortisol level of healthy people were not yet studied completely. Thus, the current study was designed and employed to assess the impact of inhalation of LEO on mood states of the healthy people using the Profile of Mood States (POMS) Questionnaire and to evaluate the changes in the salivary cortisol level during LEO exposure.

## 2. Materials and Methods

### 2.1. Preparation of *L. cubeba* Essential Oil and GC-MS Analysis

LEO was prepared by a four-hour steam distillation process as detailed by Božović et al. [16]. Fruits of *Litsea cubeba* (Lour.) Pers. were collected from Mae Hong Son Province. *L. cubeba* fruits were washed and coarsely grounded. Twenty kilograms of ground *L. cubeba* fruits were placed in the sample chamber of the distillation apparatus. The distillation apparatus is heated by gas. The generated steam passes through the sample. During the distillation process, the steam-mediated release of essential oil (vaporized form) occurs, which gets condensed while passing through the cooling system. The extracted LEO was collected and stored at 4°C in a sealed container until use.

The quantitative analysis of the chemical composition of LEO was carried out using a Gas Chromatography-Mass Spectrometer (GC-MS; GC: Perkin Elmer model Clarus 680 Waltham, MA, USA; MS: Perkin Elmer Clarus SQ8C, USA) through outsourcing at the Center for Scientific and Technological Equipment, Suranaree University of Technology. An Elite 5MS (Perkin Elmer, USA) (phase: 1,4-bis(dimethylsiloxy)phenylene dimethyl polysiloxane) capillary column with a 30 m (length) × 0.25 mm (inner diameter) and 0.25 *μ*m of film thickness was used. Helium (He) was used as the carrier gas with split ratio 1 : 20; injector temperature at 280°C, detector temperature at 300°C, and interface temperature at 320°C. The initial temperature of the column (column oven) was kept at 50°C and increased 6°C per minute until it reached 300°C and held at the final temperature for 10 minutes. The components of LEO were identified by comparing the obtained MS data with those of MS library (National Institute for Standard Technology, NIST).

All the standards used for the quantitative analysis are the commercially available pure standards (external standards) that include (-)-*β*-pinene, *β*-citral, *cis*- and *trans*-citral, citronellal, dihydromyrcenol, germacrone, limonene, linalool, pentadecane (ChromaDex, USA), and 6-methyl-5-hepten-2-one (Sigma-Aldrich, USA). Standard solutions were prepared in different concentrations (mg/ml) and injected to GC-MS. The components of LEO were determined by evaluating their total ion count (TIC) data applying external standards. The standard curve of each pure standard was plotted with TIC (*Y*-axis) versus concentration (mg/ml; *X*-axis). Sample TIC of the same retention time, which is confirmed by MS library as the same compound, was used to calculate the concentration (mg/ml) of the LOE components from the standard curve. No internal standards were used.

### 2.2. Study Participants

Fifteen healthy volunteers were enrolled for the study. The information about the study was informed to the participants. The responded volunteers have been selected based on the inclusion criteria (no previous history of allergy to essential oil and aromatic vapor, no alcohol and/or coffee consumption, no medication, and no underlying metabolic disorders) of the study. The Research Ethics committee, Faculty of Pharmacy, Chiang Mai University, approved the experimental protocols (Approval no: 35/2562 dated 21 November 2019).

All the participants were aware of the experimental procedure and informed in detail about the objective of the study and its impacts. After the complete consultation with the researcher, the participants made the signature in the test consent forms. Before the study, volunteers provided the information about the general physical and mental health condition on the experimental day and consumption of their regular meal and other related information such as time of the food consumption and satisfaction of their meal to make sure that everything is normal.

### 2.3. Measurement of the Blood Pressure and Heart Rate and Saliva Sampling

The blood pressure and heart rate of the participants were measured using an automatic dual heart rate and pressure analyzer (model no: HEM-7211, Omron Healthcare Europe B.V., The Netherlands) at three different phases of the experiment, such as the resting phase, distilled water vapor exposure, and LEO vapor exposure. Likely, saliva samples were also collected at three experimental points in sterile containers and immediately stored at −20°C until further examination.

### 2.4. Experimental Procedure

**Figure 1:**
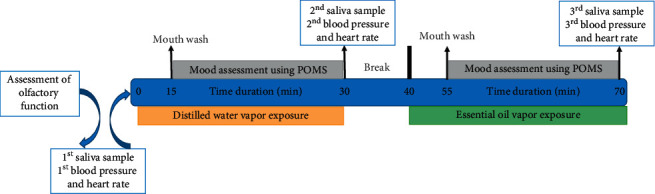
Schematic representation of study protocol.

The detailed experimental time frame is illustrated in Figure 1. Briefly, all the participants were assigned to smell the distilled water vapor and *n*-butanol (0.005%) vapor to assess the defects in their olfactory function. The volunteers those who passed the olfactory dysfunction study proceeded to further experiments.

The experiment was conducted in an air-conditioned room without any external noises and flavor. The participants were seated in a comfortable position, the first saliva sample was collected, and blood pressure and heart rates were measured (considered as resting phase samples). Then, the subjects were exposed to distilled water vapor, generated using a commercial aroma diffuser. After 15 min of distilled water vapor exposure, subjects were instructed to clean their mouth using drinking water. The POMS 2® questionnaire (developed by Heuchert J and McNair DM; Multi-Health System Inc.) was given to them and were asked to fill the questionnaire within 15 min. After 30 min of distilled water vapor exposure, the 2^nd^ saliva sample was collected, and blood pressure and heart rates were measured. Then, the subjects were allowed to take rest for 10 min, before starting the LEO vapor treatment. After the break, volunteers were exposed to LEO vapor similarly as distilled water vapor exposure, and the same sampling and measurements were performed during the LEO vapor exposure.

### 2.5. Estimation of the Salivary Cortisol Level

The salivary cortisol level of the subjects was measured using a commercial salivary cortisol ELISA kit (COR32-K01, Eagle biosciences Inc., USA) as per the manufacturer's instructions.

### 2.6. Statistical Analysis

Each experiment was performed in duplicates. Values were expressed as the mean ± standard deviation. A nonparametric test (Wilcoxon rank-sum test) was performed to assess the intragroup significance and the association of the salivary cortisol level and mood state using STATA statistical program.

## 3. Results

### 3.1. Composition of *L. cubeba* Essential Oil

The percentage yield of LEO extract obtained from *L. cubeba* fruits by steam distillation was 0.3% (w/w). The LEO composition was measured by using a GC-MS, and the results were tabulated (Table 1). (-)-*β*-Pinene, *β*-citral, *cis*- and *trans*-citral, citronellal, limonene, linalool, and 6-methyl-5-hepten-2-one were detected in LEO samples at the concentration of 4.28, 838, 936, 14.6, 68, 16, and 47.8 mg/ml of LEO, respectively. Pentadecane, germacrone, and dihydromyrcenol were not detected (Table 1).

### 3.2. Participants

The study participants were selected based on the inclusion criteria. Fifteen volunteers (5 males and 10 females) participated in the study. Eight volunteers are English-American, and seven are Asian. The demographic information of the participants is detailed in Table 2. The experiment was carried out without any interruption.

### 3.3. Heart Rate and Blood Pressure

The average heart rate of participants at resting (R), distilled water (DW) exposure, and LEO exposure was 78.00 ± 12.84, 74.86 ± 13.17, and 74.03 ±11.27 beats/min, respectively. The average heart rate differed among the male (R: 68.80 ± 6.88, DW: 65.00 ±4.76, and LEO: 64.90 ±3.80 beats/min) and female (R: 82.60 ± 12.85, DW: 80.33 ±13.28, and LEO: 78.60± 11.03 beats/min) participants. Based on the geographical subgrouping, the average heart rate of English-Americans was R: 77.50 ± 12.33, DW: 74.94 ± 11.08, and LEO: 74.44 ± 11.54 beats/min, while Asians showed the average heart rate of R: 78.57 ± 14.38, DW: 74.75 ± 16.71, and LEO: 73.57 ± 11.86 beats/min (Table 3).

The average blood pressure (systolic/diastolic) of participants at resting (R), distilled water (DW) exposure, and LEO exposure was 111.53 ± 10.4666.43 ± 6.55, 107.32 ± 12.22/62.57 ± 6.38, and 107.57 ± 11.8463.50 ± 7.05 mmHg, respectively.

The average blood pressure (systolic/diastolic) of male participants at resting (R), distilled water (DW) exposure, and LEO exposure was 120.20 ± 10.09/67.60 ± 4.62, 118.60; ± 12.74/62.00; ± 4.81, and 119.80; ± 10.3966.40 ± 7.55 mmHg, respectively. The average blood pressure (systolic/diastolic) of female participants at resting (R), distilled water (DW) exposure, and LEO exposure was 107.20 ± 7.90/65.85 ± 7.49, 101.06 ± 6.14/62.89 ± 7.37, and 101.45 ± 6.74/62.05 ± 6.70 mmHg, respectively.

The average blood pressure (systolic/diastolic) of English-American and Asian participants at resting (R), distilled water (DW) exposure, and LEO exposure was 117.31 ± 8.79/68.38 ± 6.11 and 104.93 ± 8.36/64.21 ± 6.76, 112.62 ± 13.05/64.31 ± 6.41 and 100.25 ± 6.68/60.25 ± 6.08, and 113.88 ± 10.48/66.13 ± 7.29 and 100.36 ± 9.23/60.50 ± 5.84 mmHg, respectively (Table 3).

### 3.4. Change in the Mood Status

The average total mood disturbance (TMD), hostility/anger, confusion, depression, fatigue, tension, and vigor during DW exposure were 18.33 ± 22.36, 1.80 ± 2.04, 9.67 ± 5.22, 5.40 ± 5.58, 6.60 ± 5.60, 8.47 ± 6.69, and 13.60 ± 6.19, respectively. The average total mood disturbance (TMD), hostility/anger, confusion, depression, fatigue, tension, and vigor during LEO exposure were−1.53 ± 14.17, 0.53 ± 0.83, 6.00 ± 3.30, 1.87±2.17, 3.27± 2.43, 2.80 ± 2.54, and 13.87 ± 7.74, respectively (Table 4).

The mean score change of mood states between male and female participants was measured. The male subjects showed the differences of −9.40 ± 9.45, −1.60 ± 2.07, −0.40 ± 2.30, −2.80 ± 4.66, −0.80 ± 1.64, −4.20 ± 3.11, and −0.40 ± 5.68 for TMD, hostility/anger, confusion, depression, fatigue, tension, and vigor, respectively. The female subjects showed the differences of −25.10 ± 16.68, −1.10 ± 1.29, −5.30 ± 4.00, −3.90 ± 3.21, −4.60 ± 3.98, −6.40 ± 7.41, and −0.60 ± 9.69 for TMD, hostility/anger, confusion, depression, fatigue, tension, and vigor, respectively. The TMD (*p*=0.0365) and confusion (*p*=0.0160) values showed significant difference between male and female subjects (Table 5).

The mean score change of mood states of English-Americans for TMD, hostility/anger, confusion, depression, fatigue, tension, and vigor was −18.13 ±10.03, −1.38± 1.69, −2.50 ± 3.07,−3.25 ± 3.85,−3.00  ± 2.93,−5.88 ±  2.95, and 2.13±6.42, respectively. The mean score change of mood states of Asian participants for TMD, hostility/anger, confusion, depression, fatigue, tension, and vigor was −21.86  ± 22.08,−1.14 ± 1.46,−5.00 ± 5.09,−3.85 ± 3.63, −3.71 ± 4.82, −5.43 ± 9.02, and −1.86  ± 10.24, respectively. None of the parameters (TMD (*p*=0.6841), hostility/anger (*p*=0.7620),confusion(*p*=0.3209),depression (*p*=0.7256), fatigue (*p*=1.00), tension (*p*=0.2690), and vigor (*p*=0.4499)) showed the significant difference among the groups (Table 6).

### 3.5. Change in the Cortisol Level

The average salivary cortisol level of the participants during resting/baseline (R), distilled water (DW) exposure, and LEO exposure was 1.49 ± 0.73, 1.04 ± 0.50, and 0.79 ± 0.35 ng/ml, respectively. The mean change from the baseline value of salivary cortisol during DW exposure and LEO exposure was −0.45 ± 0.50 and −0.70 ± 0.13, respectively. The male subjects showed R: 1.20 ± 0.88, DW: 0.91 ± 0.62, and LEO: 0.70 ± 0.53 ng/ml of salivary cortisol, while female volunteers displayed R: 1.63 ± 0.65, DW: 1.10 ± 0.46, and LEO: 0.83 ± 0.25 ng/ml of salivary cortisol. The salivary cortisol level of English-American participants was R: 1.43 ± 0.68, DW: 0.88 ± 0.49, and LEO: 0.72 ± 0.38 ng/ml, and the Asian subjects showed the salivary cortisol level of R: 1.55 ± 0.85, DW: 1.23 ± 0.48, and LEO: 0.86 ± 0.33 ng/ml (Table 7). The correlation between the change in salivary cortisol and mood states of the volunteers during LEO exposure was not significant (Table 8).

## 4. Discussion

LEO was extracted, and the composition of LEO was determined by gas chromatography/mass spectrometry (GC-MS). The results showed that LEO was rich in citral, limonene, 6-methyl-5-hepten-2-one, and linalool (Table 1). The observation was supported by previous reports. For instance, citral B (neral), *β*-phellandrene, and *β*-terpinene are the major compounds found in essential oils extracted from several parts of the *L. cubeba* plant [7]. GC/MS analysis showed that LEO is composed of 23 compounds, including geranial, neral, and *d*-limonene [17]. Thielmann and Muranyi [18] reviewed the chemical composition of LEO and stated that citral and limonene are the most predominant compounds present in the *L. cubeba* fruit essential oil. Hammid and Ahmad [19] investigated the chemical compositions of the compounds present in the several parts (bark, fruit, leaf, root, and stem) of the *L. cubeba* plant by GC-MS analysis. Monoterpenes (citronellal, D-limonene, citronellol, *trans*-geraniol, isopulegol, *β*-linalool, *β*-myrcene, *α*-citral eucalyptol, *cis*-citral, *α*-terpineol, and melon heptanal) and sesquiterpenes (terpin hydrate, citronellic acid, *α*-caryophyllene, and caryoplyllene oxide) were the compounds identified in the essential oil extracted from *L. cubeba* fruit [19]. The combined GC/MS and headspace-solid phase microextraction analysis showed that LEO consists of 56 components. Specifically, monoterpenes, sesquiterpenes, terpene alcohols, terpene aldehydes, terpene ketone, terpene ester, terpene oxide, aliphatic aldehydes, aliphatic alcohols, aliphatic ketone, and aliphatic ester are the major compounds found in *L. cubeba* fruit essential oil [20].

LEO showed immunosuppressive activity. More specifically, LEO suppressed the production of IL-12 and TNF-*α* in a dose-dependent manner in endotoxin-induced dendritic cells and inhibited the hypersensitivity reaction and T-cell infiltration in mice. The results demonstrated that LEO exhibits immunosuppressive ability [20].

LEO supplementation improved the sleeping time in the pentobarbitone-induced mouse in dose-dependent manner. LEO supplementation reduced the anxiety in experimental mice. *L. cubeba* oil-treated mice spend more time in open arm and made higher open arm entry in an elevated plus maze study when compared to control animals. LEO supplementation (500 mg/kg L) showed potent analgesic activity. The results suggested that LEO has potent neuroprotective activity [17].

LEO isolated from different parts of the plant exhibited moderate antimicrobial activity against *Bacillus subtilis*, *Escherichia coli*, *Enterococcus faecalis*, *Monilia albicans*, *Pseudomonas aeruginosa*, and *Staphylococcus aureus* [7]. LEO exhibited antimicrobial activity against methicillin-resistant *Staphylococcus aureus*. The disruption of the cell membrane, inhibition of microbial virulence enzyme, and suppression of the hexose monophosphate pathway were attributed to the antimicrobial activity of LEO [9].

In this study, healthy subjects were exposed to LEO vapor, and the changes in mood state were analyzed by the POMS 2® questionnaire and salivary cortisol level by ELISA. The change in the average heart rate during LEO exposure was not significant in this study when compared to both R, and DW exposure. Bergamot essential oil exposure increased the high-frequency heart rate value in healthy subjects [21]. In the present study, female subjects showed a relatively high average heart rate at the R stage (82.60 ± 12.85 vs. 68.80 ± 6.88) compared to that of the male participants. The overall and subgroup analysis results suggested that the average heart rate was reduced during DW and LEO exposure compared to the R value, but not at a significant level (Table 3). The change in blood pressure (both systolic and diastolic) was not significant between treatments. The subgrouping of subjects showed a notable difference in systolic pressure among male and female subjects and English-American and Asian subjects, but these variations did not hinder the study results (Table 3).

Previous studies suggested that exposure to essential oil could reduce the cortisol level in healthy subjects. For instance, the exposure to the aroma of essential oil of lavender does not significantly alter the salivary cortisol level in healthy volunteers, who performed a serial arithmetic task before the study. The level of salivary chromogranin A was increased after the arithmetic task and reduced after the resting period during lavender essential oil exposure. The results recommended that exposure to lavender aroma could reduce arithmetic task-induced stress [22]. Similarly, the exposure to lavender and rosemary oil reduced the oxidative stress and improved the free radical scavenging activity of the host in healthy volunteers. Particularly, the cortisol level was reduced after lavender and rosemary oil exposure, while no changes were observed in the salivary IgA and *α*-amylase level [23]. Ahmad et al. [24] conducted a single-blind, randomized, placebo-controlled trial in male pharmacy students to evaluate the effect of aromatherapy with lavender oil on academic stress during the exams and concluded that academic stress was not improved by the aromatherapy with lavender oil. Hamzeh et al. [25] conducted a randomized controlled trial to investigate the effect of aromatherapy with lavender essential oil and peppermint essential oil on the sleep quality of cancer patients. Exposure to essential oil (lavender essential oil or peppermint essential oil) reduced the Pittsburgh Sleep Quality Inventory (PSQI) in the cancer patients of the intervention group compared to that of the control group indicating that aromatherapy could improve the quality of sleep of cancer patients [25].

Aromatherapy reduced the salivary cortisol level and stress in middle school students effectively. The study concluded that aroma essential oil therapy reduces the stress and aids in health management in a modern competitive society and could be used as a complementary and alternative therapy for wellbeing [26].

Watanabe et al. [21] studied the effect of bergamot essential oil exposure on mood states, parasympathetic nervous activity, and salivary cortisol levels in healthy female volunteers. The subjects were exposed to essential oil for 15 min, and State-Trait Anxiety Inventory and Fatigue Self-Check List and Profile of Mood States and salivary cortisol were assessed. The results showed that the salivary cortisol level varied at resting, water vapor exposure, and essential oil exposure, and specifically during essential oil exposure, the cortisol level was significantly low when compared to the resting value. The results suggested that bergamot essential oil exposure helps to improve the emotions and reduce the stress in healthy humans.

In the present study, LEO inhalation significantly improved the total mood disturbance, especially reduced the confusion among the subjects (Table 5). The positive effect of LEO vapor inhalation was not altered among the subjects in terms of ethnic variation (Table 6). The salivary cortisol level was reduced during LEO inhalation when compared to distilled water exposure and baseline values, whereas Spearman's rank correlation showed that the change in salivary cortisol and mood states of the volunteers during LEO exposure was not significant (Table 8).

## 5. Conclusions

The inhalation of LEO significantly improved the total mood disturbance and reduced the confusion among the healthy human subjects. LEO inhalation reduced the salivary cortisol level at a notable level. The study results indicated that aromatherapy with *L. cubeba* fruit essential oil could improve the mood states and reduce stress and could be used as a complementary and alternative therapy for wellbeing. The results of the current study warrant further studies on the beneficial effect of LEO aromatherapy in healthy and diseased subjects to uncover the therapeutic nature of the *L. cubeba* plant.

## Figures and Tables

**Table 1:** Chemical composition of *L. cubeba* essential oil.

Constituents	*Litsea cubeba* oil (mg/ml)
Pentadecane	Not detected
(-)-beta-Pinene	4.28
beta-Citral	838
Cis- and trans-citral	936
Citronellal	14.6
Germacrone	Not detected
Limonene	68.0
Linalool	16.0
Dihydromyrcenol	Not detected
6-Methyl-5-hepten-2-one	47.8

**Table 2:** Demographic data of the participants.

Sample group	Quantity	Percentage
Total	15	100

Sex	15	100
Male	5	33.33
Female	10	66.67

Age (year)	15	100
21–30	13	86.67
31–40	2	13.33

Nationality	15	100
British-American	8	53.33
(i) British	1	6.67
(ii) American	7	46.67
Asian	7	46.67
(i) Vietnam	1	6.67
(ii) China	1	6.67
(iii) Turkmenistan	1	6.67
(iv) Indie	1	6.67
(v) Brunei	3	20.00

BMI (kg/m^2^)	15	100
18.5–24.9	13	86.66
25.0–29.9	1	6.67
≥30.0	1	6.67

Education	15	100
High school	5	33.33
Bachelor's degree	7	46.67
Master's degree	2	13.33
Doctorate	1	6.67

**Table 3:** Changes in the heat rate and blood pressure during the inhalation of essential oil.

Parameters	*n*	Values (mean ± SD)		
		Rest	Distilled water	Essential oil
*Heart rate (beats/min)*				
Total	15	78.00 ± 12.84	74.86 ± 13.17	74.03 ± 11.27
Gender				
Male	5	68.80 ± 6.88	65.00 ± 4.76	64.9 ± 3.80
Female	10	82.60 ± 12.85	80.33 ± 13.28	78.6 ± 11.03
Nationality				
English-American	8	77.50 ± 12.33	74.94 ± 11.08	74.44 ± 11.54
Asian	7	78.57 ± 14.38	74.75 ± 16.71	73.57 ± 11.86

*Systolic blood pressure (mmHg)*				
Total	15	111.53 ± 10.46	107.32 ± 12.22	107.57 ± 11.84
Gender				
Male	5	120.20 ± 10.09	118.60 ± 12.74	119.80 ± 10.39
Female	10	107.20 ± 7.90	101.06 ± 6.14	101.45 ± 6.74
Nationality				
English-American	8	117.31 ± 8.79	112.62 ± 13.05	113.88 ± 10.48
Asian	7	104.93 ± 8.36	100.25 ± 6.68	100.36 ± 9.23

*Diastolic blood pressure (mmHg)*				
Total	15	66.43 ± 6.55	62.57 ± 6.38	63.50 ± 7.05
Gender				
Male	5	67.60 ± 4.62	62.00 ± 4.81	66.40 ± 7.55
Female	10	65.85 ± 7.49	62.89 ± 7.37	62.05 ± 6.70
Nationality				
English-American	8	68.38 ± 6.11	64.31 ± 6.41	66.13 ± 7.29
Asian	7	64.21 ± 6.76	60.25 ± 6.08	60.50 ± 5.84

**Table 4:** Changes in mood states during the inhalation of essential oil.

Mood states	*n*	Scores (mean ± SD)	
		Distilled water	Essential oil
*Total mood disturbance*			
Total	15	18.33 ± 22.36	−1.53 ± 14.17
Gender			
Male	5	9.00 ± 17.29	−0.40 ± 15.82
Female	10	23.00 ± 23.93	−2.10 ± 14.14
Nationality			
English-American	8	16.00 ± 14.66	−2.13 ± 13.77
Asian	7	21.00 ± 30.01	−0.86 ± 15.69

*Hostility/anger*			
Total	15	1.80 ± 2.04	0.53 ± 0.83
Gender			
Male	5	2.20 ± 2.17	0.60 ± 0.89
Female	10	1.60 ± 2.07	0.50 ± 0.85
Nationality			
English-American	8	1.88 ± 1.89	0.50 ± 0.76
Asian	7	1.71 ± 2.36	0.57 ± 0.98

*Confusion*			
Total	15	9.67 ± 5.22	6.00 ± 3.36
Gender			
Male	5	6.40 ± 1.95	6.00 ± 4.18
Female	10	11.3 ± 5.64	6.00 ± 3.13
Nationality			
English-American	8	8.38 ± 2.92	5.88 ± 3.44
Asian	7	11.14 ± 6.99	6.14 ± 3.53

*Depression*			
Total	15	5.40 ± 5.58	1.87 ± 2.17
Gender			
Male	5	4.60 ± 7.54	1.80 ± 2.95
Female	10	5.80 ± 4.76	1.90 ± 1.85
Nationality			
English-American	8	5.00 ± 6.12	1.75 ± 2.38
Asian	7	5.86 ± 5.34	2.00 ± 2.08

*Fatigue*			
Total	15	6.60 ± 5.60	3.27 ± 2.43
Gender			
Male	5	3.40 ± 3.44	2.60 ± 2.61
Female	10	8.20 ± 5.92	3.60 ± 2.41
Nationality			
English-American	8	6.38 ± 3.89	3.38 ± 2.00
Asian	7	6.86 ± 7.45	3.14 ± 3.02

*Tension*			
Total	15	8.47 ± 6.69	2.80 ± 2.54
Gender			
Male	5	7.20 ± 5.12	3.00 ± 3.16
Female	10	9.10 ± 7.52	2.70 ± 2.36
Nationality			
English-American	8	8.00 ± 4.07	2.13 ± 2.64
Asian	7	9.00 ± 9.18	3.57 ± 2.37

*Vigor*			
Total	15	13.60 ± 6.19	13.87 ± 7.74
Gender			
Male	5	14.80 ± 4.32	14.40 ± 7.30
Female	10	13.00 ± 7.07	13.60 ± 8.32
Nationality			
English-American	8	13.63 ± 6.37	15.75 ± 7.81
Asian	7	13.57 ± 6.48	11.71 ± 7.63

**Table 5:** Means score change from baseline (inhalation of distilled water) of mood states, comparing male and female.

Mood states	Mean change from base line ± std. deviation		*p* value
	Male	Female	
TMD	−9.40 ± 9.45	−25.10 ± 16.68	0.0365^*∗*^
Anger	−1.60 ± 2.07	−1.10 ± 1.29	0.7486
Confusion	−0.40 ± 2.30	−5.30 ± 4.00	0.0160^*∗*^
Depression	−2.80 ± 4.66	−3.90 ± 3.21	0.3531
Fatigue	−0.80 ± 1.64	−4.60 ± 3.98	0.0731
Tension	−4.20 ± 3.11	−6.40 ± 7.41	0.8055
Vigor	−0.40 ± 5.68	0.60 ± 9.69	0.4239

**Table 6:** Means score change from baseline (inhalation of distilled water) of mood states, comparing between nationalities.

Mood states	Mean change from base line ± std. deviation		*p* value
	British-American	Asian	
TMD	−18.13 ± 10.03	−21.86 ± 22.08	0.6841
Anger	−1.38 ± 1.69	−1.14 ± 1.46	0.7620
Confusion	−2.50 ± 3.07	−5.00 ± 5.09	0.3209
Depression	−3.25 ± 3.85	−3.85 ± 3.63	0.7256
Fatigue	−3.00 ± 2.93	−3.71 ± 4.82	1.0000
Tension	−5.88 ± 2.95	−5.43 ± 9.02	0.2690
Vigor	2.13 ± 6.42	−1.86 ± 10.24	0.4499

**Table 7:** Change in mean salivary cortisol in volunteers.

Group	Baseline (ng/ml)	Distilled water inhalation (ng/ml)		Essential oil inhalation (ng/ml)	
		Mean ± SD	Mean change from baseline ± SD	Mean ± SD	Mean change from baseline ± SD
Total	1.49 ± 0.73	1.04 ± 0.50	−0.45 ± 0.50	0.79 ± 0.35	−0.70 ± 0.13

Gender					
Male	1.20 ± 0.88	0.91 ± 0.62	−0.29 ± 0.35	0.70 ± 0.53	−0.51 ± 0.66
Female	1.63 ± 0.65	1.10 ± 0.46	−0.53 ± 0.56	0.83 ± 0.25	−0.80 ± 0.51

Nationality					
English-American	1.43 ± 0.68	0.88 ± 0.49	−0.55 ± 0.32	0.72 ± 0.38	−0.71 ± 0.35
Asian	1.55 ± 0.85	1.23 ± 0.48	−0.33 ± 0.66	0.86 ± 0.33	−0.69 ± 0.76

**Table 8:** Spearman's rank correlation between change in salivary cortisol and mood states.

Parameters		Mood states						
		TMD	Anger	Confusion	Depression	Fatigue	Tension	Vigor
Change in salivary cortisol	Rho	0.0548	0.4687	−0.1614	0.4544	−0.1213	−0.1650	−0.1213
	*p* values	0.8463	0.0780	0.5655	0.0888	0.6667	0.5567	0.6667

## Data Availability

All relevant data are included in the manuscript.

## References

[1] Cheraghbeigi N., Modarresi M., Rezaei M., Khatony A. (2019). Comparing the effects of massage and aromatherapy massage with lavender oil on sleep quality of cardiac patients: a randomized controlled trial. *Complementary Therapies in Clinical Practice*.

[2] Heydarirad G., Keyhanmehr A. S., Mofid B., Nikfarjad H., Mosavat S. H. (2019). Efficacy of aromatherapy with *Rosa damascena* in the improvement of sleep quality of cancer patients: a randomized controlled clinical trial. *Complementary Therapies in Clinical Practice*.

[3] Wang Y.-S., Wen Z.-Q., Li B.-T., Zhang H.-B., Yang J.-H. (2016). Ethnobotany, phytochemistry, and pharmacology of the genus Litsea: an update. *Journal of Ethnopharmacology*.

[4] Agrawal N., Choudhary A. S., Sharma M. C., Dobhal M. P. (2011). Chemical constituentsof plants from the *Genus litsea*. *Chemistry & Biodiversity*.

[5] Li Y., Kong W., Li M. (2016). *Litsea cubeba* essential oil as the potential natural fumigant: inhibition of *Aspergillus flavus* and AFB1 production in licorice. *Industrial Crops and Products*.

[6] Huang X.-W., Feng Y.-C., Huang Y., Li H.-L. (2013). Potential cosmetic application of essential oil extracted from *Litsea cubeba* fruits from China. *Journal of Essential Oil Research*.

[7] Wang H., Liu Y. (2010). Chemical composition and antibacterial activity of essential oils from different parts of *Litsea cubeba*. *Chemistry & Biodiversity*.

[8] Si L., Chen Y., Han X. (2012). Chemical composition of essential oils of *Litsea cubeba* harvested from its distribution areas in China. *Molecules*.

[9] Hu W., Li C., Dai J., Cui H., Lin L. (2019). Antibacterial activity and mechanism of Litsea cubeba essential oil against methicillin-resistant *Staphylococcus aureus* (MRSA). *Industrial Crops and Products*.

[10] Li W. R., Shi Q. S., Liang Q., Xie X. B., Huang X. M., Chen Y. B. (2014). Antibacterialactivity and kinetics of litsea cubeba oil on *Escherichia coli*. *PLoS One*.

[11] Liao P.-C., Yang T.-S., Chou J.-C. (2015). Anti-inflammatory activity of neral and geranial isolated from fruits of *Litsea cubeba* Lour. *Journal of Functional Foods*.

[12] Seal S., Chatterjee P., Bhattacharya S. (2012). Vapor of volatile oils from *Litsea cubeba* seed induces apoptosis and causes cell cycle arrest in lung cancer cells. *PLoS One*.

[13] Hwang J.-K., Choi E.-M., Lee J. H. (2005). Antioxidant activity of *Litsea cubeba*. *Fitoterapia*.

[14] Jiang Z., Akhtar Y., Bradbury R., Zhang X., Isman M. B. (2009). Comparative toxicity of essential oils of *Litsea pungens* and *Litsea cubeba* and blends of their major constituents against the cabbage looper, *Trichoplusia ni*. *Journal of Agricultural and Food Chemistry*.

[15] Ali B., Al-Wabel N. A., Shams S., Ahamad A., Khan S. A., Anwar F. (2015). Essential oils used in aromatherapy: a systemic review. *Asian Pacific Journal of Tropical Biomedicine*.

[16] Božović M., Navarra A., Garzoli S., Pepi F., Ragno R. (2017). Essential oils extraction: a 24-hour steam distillation systematic methodology. *Natural Product Research*.

[17] Chen C.-J., Tseng Y.-H., Chu F.-H. (2012). Neuropharmacological activities of fruit essential oil from *Litsea cubeba* Persoon. *Journal of Wood Science*.

[18] Thielmann J., Muranyi P. (2019). Review on the chemical composition of *Litsea cubeba* essential oils and the bioactivity of its major constituents citral and limonene. *Journal of Essential Oil Research*.

[19] Hammid S. A., Ahmad F. (2015). Chemotype of *Litsea cubeba* essential oil and its bioactivity. *Natural Product Communications*.

[20] Chen H.-C., Chang W.-T., Hseu Y.-C. (2016). Immunosuppressive effect of *Litsea cubeba* L. essential oil on dendritic cell and contact hypersensitivity responses. *International Journal of Molecular Sciences*.

[21] Watanabe E., Kuchta K., Kimura M., Rauwald H. W., Kamei T., Imanishi J. (2015). Effects of bergamot (*Citrus bergamia* (Risso) wright & Arn.) essential oil aromatherapy on mood states, parasympathetic nervous system activity, and salivary cortisol levels in 41 healthy females. *Forsch Komplementmed*.

[22] Toda M., Morimoto K. (2008). Effect of lavender aroma on salivary endocrinological stress markers. *Archives of Oral Biology*.

[23] Atsumi T., Tonosaki K. (2007). Smelling lavender and rosemary increases free radical scavenging activity and decreases cortisol level in saliva. *Psychiatry Research*.

[24] Ahmad R., Naqvi A. A., Al-Bukhaytan H. M., Al-Nasser A. H., Al-Ebrahim A. H. B. (2019). Evaluation of aromatherapy with lavender oil on academic stress: a randomized placebo controlled clinical trial. *Contemporary Clinical Trials Communications*.

[25] Hamzeh S., Safari-Faramani R., Khatony A. (2020). Effects of aromatherapy with lavender and peppermint essential oils on the sleep quality of cancer patients: a randomized controlled trial. *Evidence-Based Complementary and Alternative Medicine*.

[26] Won S.-J., Choi Y.-S. (2017). The effect of aromatherapy upon salivary cortisol, academic stress, academic self-efficacy and suicidal ideation in middle school students. *Biomedical Research*.

